# Sociodemographic, Economic, and Health Factors Associated with Ultra-Processed Food Intake Among Older Adults in Chile

**DOI:** 10.3390/nu18121899

**Published:** 2026-06-12

**Authors:** Daiana Quintiliano-Scarpelli, Leticia de Albuquerque Araújo, Camila Zancheta Ricardo

**Affiliations:** 1 Nutrition and Dietetics Program, Faculty of Medicine, Clínica Alemana–Universidad del Desarrollo, Avenida La Plaza 680, Las Condes, Santiago 7610658, Chile; 2 Nutrition and Dietetics School, Faculty of Health Sciences, Catholic University of Maule, Curicó 3340000, Chile; 3 Center of Research in Food Environment and Prevention of Obesity and Non-Communicable Diseases (CIAPEC), Institute of Nutrition and Food Technology (INTA), University of Chile, Santiago 7830490, Chile

**Keywords:** ultra-processed foods, NOVA classification, older adults, sociodemographic factors, dietary intake

## Abstract

**Background/Objectives:** Consumption of ultra-processed foods (UPF) has been linked to poorer diet quality and adverse health outcomes. Although Chile ranks among the highest consumers of UPFs in Latin America, studies using primary dietary data, especially among older adults, are scarce. This study aimed to describe the food intake of Chilean older adults according to the degree of food processing, and to explore the association between UPF intake and sociodemographic, economic and health factors. **Methods:** A cross-sectional study of 434 non-institutionalized older adults (≥60 years) living in the Metropolitan Region of Chile was conducted. Dietary intake was assessed using interviewer-administered 24h recall, with a second assessment 8–15 days later in a random subsample (*n* = 60). Foods were classified according to the NOVA system into minimally processed foods (MPFs), culinary ingredients, processed foods (PF), or UPF. Usual energy intake was estimated using the MSM. Sociodemographic (sex, age, area), economic (income, education, health system), and health-related variables (chronic conditions, sedentary lifestyle, tobacco use) were collected through home-visit questionnaires. Anthropometric and functional measurements were taken by trained nutritionists. The association between UPF intake and studied variables was evaluated using multivariate fractional probit regression, with mean marginal effects presented. **Results:** Most of the participants were women (86.2%), aged 70–79 years (47.9%), and residents of urban areas (76.3%). Most of their calories came from MPF (45.7%), followed by PF (25.5%) and UPF (16.6%). Higher UPF intake was associated with living in an urban area (+3.8%; 95% CI 1.2–6.3%), higher education (+3.5%; 95% CI 1.1–6.0%), and being affiliated with the private health system (+9.1%; 95% CI 4.1–14.0%). **Conclusions:** In this community-based sample of Chilean older adults, UPF intake was associated with socioeconomic factors but not health status.

## 1. Introduction

In recent decades, global dietary patterns have shifted from traditional diets toward those increasingly based on industrially produced foods [1]. The NOVA food classification system, which categorizes foods based on the extent and purpose of industrial processing rather than specific nutritional content, has facilitated research on the impact of processed food consumption on health. NOVA classifies all foods and beverages into four mutually exclusive groups: (i) unprocessed or minimally processed foods, which include whole foods or parts of foods that are either unmodified or have undergone minimal processing to facilitate preservation, storage, or consumption (e.g., fresh fruits, vegetables, grains, meat, milk); (ii) processed culinary ingredients, which are substances extracted from nature or collected and used in food preparation (e.g., salt, sugar, butter, oils, vinegars); (iii) processed foods, which are products made by combining unprocessed or minimally processed foods with culinary ingredients (e.g., canned meat and vegetables, cheeses, artisan breads, fruit compotes); and (iv) ultra-processed foods (UPF), which are industrial formulations composed of ingredients—many exclusive to the food industry—combined with food additives through a series of industrial processes (e.g., soft drinks, confectionery, sausages, frozen pizzas and meals, instant soups, cookies, cakes, sandwich breads) [2].

The increasing global prevalence of UPF intake has become a significant public health concern, as higher intake of these products is associated with poorer nutritional quality, characterized by higher energy density, excessive free sugars, and saturated fats, and lower fiber content [3]. Additionally, UPF intake has been associated with multiple adverse health outcomes, including obesity, cardiovascular diseases, type 2 diabetes, and certain types of cancer [4]. These associations are particularly concerning in the elderly population, who face additional vulnerabilities due to physiological, functional, psychological, social, and economic factors [5,6].

The proportion of people aged 65 years and older worldwide has increased significantly, from 6% in 1990 to 9% in 2020, and is projected to reach 16% by 2050 [7]. In Latin America, Chile has the highest life expectancy at birth, increasing from 77 years in 2000 to 80.7 years by 2025 [8]. Additionally, projections from the National Institute of Statistics (INE) and the Economic Commission for Latin America and the Caribbean (CEPAL) indicate that Chile will be the third country in the region with the highest old-age dependency ratio, reaching an index of 21.2 by 2025 [8,9].

Despite these demographic achievements, older adults in Chile face multiple challenges related to dietary intake. An analysis based on the most recent National Health Survey (2016–2017) found that most older adults in Chile suffer from multimorbidity, defined as the presence of two or more non-communicable diseases (NCD) (63.2%), and only 3.9% satisfactorily complied with dietary guidelines concerning the intake of fruits, vegetables, fish, legumes, water, and dairy products [10]. Research on UPF intake among older adults in Chile remains scarce. To the best of our knowledge, only one estimate on the intake of UPF among older adults has been conducted in Chile based on the 2010 National Food Intake Survey (ENCA). Cediel et al. reported that 18.3% of the total energy intake of older Chilean adults came from UPFs, a lower proportion compared to the general population (28.6%) [11]. However, these findings may not accurately reflect current consumption patterns, especially given changes in food environments, aging demographics, and dietary habits over the past decade. Moreover, previous studies have not examined sociodemographic, economic, and health factors associated with UPF intake in this age group, nor accounted for urban-rural disparities. Urban areas, with a higher density of food outlets and greater accessibility to UPF, have been associated with poorer diet quality [12,13], potentially exacerbating NCD in this population [14]. In contrast, rural areas often present geographical and economic barriers that limit access to fresh foods, resulting in dietary patterns shaped by local availability and self-sufficiency strategies [15]. Understanding these differences is critical for designing equitable public health interventions, as urban and rural older adults face distinct structural barriers to healthy eating. In this context, the present study aims to describe dietary intake according to the NOVA classification among older adults residing in the Metropolitan Region of Chile and to explore association of ultra-processed food intake with sociodemographic, economic, and health factors.

## 2. Materials and Methods

### 2.1. Study Design and Participants

This cross-sectional, observational, and community-based analytical study was conducted as part of the Fondecyt Initiation Project No. 11220503, funded by the National Agency for Research and Development (ANID). The sample size was calculated to test the hypothesis of differences in dietary quality according to the area of residence using a multiple regression model (F-test for R-squared), with 80% statistical power, a 5% significance level, and an estimated R-squared of 0.05. The required sample size was 363 individuals, which was increased by 20% to account for potential losses. The final sample was stratified into urban and rural zones based on the rurality index and distributed proportionally to the population size in each area.

The selected study area included eight municipalities from three provinces (Santiago, Melipilla, and Talagante) within the Metropolitan Region of Santiago, Chile. This region was chosen due to its feasibility within the broader research framework. It is the largest and most densely populated region in the country, comprising 52 municipalities across six provinces. The selected municipalities represent distinct levels of urbanization while sharing similar socioeconomic characteristics and levels of social priority [16].

Inclusion criteria comprised individuals aged 60 years or older, non-institutionalized, with normal cognitive function (Mini-Mental State Examination score > 14) [17], and able to consume food orally. Participants were recruited through municipal offices and senior clubs in the selected municipalities (Cerro Navia, Renca, Lo Prado, Pudahuel, Melipilla, Talagante, Peñaflor, and El Monte), with support from municipal service directors. Interviews were conducted either at the participants’ homes or at the municipal club facilities, depending on their preference and convenience.

A total of 519 individuals were recruited; however, interviews could not be conducted with 79 participants due to acute illness or hospitalization (*n* = 27), inability to be contacted after multiple attempts (*n* = 18), scheduling conflicts (*n* = 25), and refusal to participate (*n* = 9). The final sample consisted of 434 individuals, of whom 331 resided in urban areas and 103 in rural areas. Data collection was conducted between December 2022 and September 2023.

The study was approved by the Ethics Committee of the School of Medicine at Universidad del Desarrollo-Clínica Alemana (protocol number 2021-237; approved on 7 March 2022), and all participants provided written informed consent prior to participation. This study was reported using the Strengthening the Reporting of Observational Studies in Epidemiology (STROBE) [18].

### 2.2. Dietary Data

Trained nutritionists conducted face-to-face 24 h dietary recalls (24h-DR) following the United States Department of Agriculture (USDA) Automated Multiple-Pass method [19]. To ensure accurate portion size estimation, we employed the Photographic Atlas of Foods and Typical Preparations of Chile, validated in the National Food Intake Survey of Chile [20]. Dietary data was entered into SER-24, software developed by the Institute of Nutrition and Food Technology (INTA, University of Chile, Santiago, Chile) that uses nutritional content from the USDA Food Composition Databases [21].

All foods and beverages consumed were categorized according to the NOVA classification in group 1: unprocessed or minimally processed foods; group 2: processed culinary ingredients; group 3: processed foods; and group 4: UPF. The classification was conducted according to FAO guidelines [2], with two trained researchers performing the classification independently. The interrater reliability was assessed using Cohen’s kappa coefficient (κ = 0.80), and disagreements were resolved by discussion with the authors. A total of 1368 foods were identified, of which 36.4% (*n* = 498) were classified as group 1, 10.9% (*n* = 149) as group 2, 6.8% (*n* = 93) as group 3, and 45.9% (*n* = 628) as group 4. Because a single 24h-DR does not adequately represent usual daily intake, and conducting multiple measurements for the entire sample would impose high resource demands and participant burden, we applied a validated statistical method to estimate the usual intake based on repeated measurements from a subsample [22]. A second non-consecutive 24h DR was collected using the same face-to-face procedure within an interval of 8 to 15 days in a random subsample of 60 participants. The subsample size was determined based on the minimal number of 50 participants recommended for this analysis [23]. We used the Multiple Source Method (MSM), version 1.0.2e (German Institute of Human Nutrition Potsdam-Rehbrücke, Nuthetal, Germany), a statistical tool available online for estimating usual dietary intake [24], which applies a two-part regression model to adjust for intraindividual variability (i.e., differences in intake on different days by the same individual) and interindividual variability (i.e., differences between individuals). This method was implemented to estimate the usual percentage of calories consumed from each NOVA group, incorporating age and sex as covariates.

### 2.3. Sociodemographic, Economic, and Health Data

Collected data included information on socioeconomic characteristics, health conditions, and lifestyle factors obtained through structured surveys. Anthropometric measurements were performed following the protocols established by the National Health and Nutrition Examination Survey [25].

Among the sociodemographic variables, sex (male or female) and age (in years) were recorded. Household composition was classified into two categories: living alone or living with family (including (i) partner, (ii) children and grandchildren, and (iii) other configurations such as parents, in-laws, siblings, son- or daughter-in-law, other relatives, paid or unpaid domestic workers, or caregivers). The zone of residence was classified as urban or rural, based on the national criteria established by the Natural Resources Information Center [26]. Economic variables included educational level, categorized as less than secondary education (including no formal education, incomplete basic education, complete basic education, or incomplete secondary education), complete secondary, and higher education. Household income was classified into four levels: less than one minimum wage (reference value: US$365), 1 to 2 minimum wages, and more than 3 minimum wages. Health system affiliation was categorized as public or private. Food insecurity was assessed using the Food Insecurity Experience Scale (FIES) and analyzed with the Rasch model according to FAO guidelines [27]. Standard global cut-points (ATELESS for moderate/severe and WHLDAY for severe) were applied to estimate individual probabilities, and population prevalences were derived as weighted means.

Health variables included the number of NCDs, obtained through self-report, including diabetes, arterial hypertension, heart failure, acute myocardial infarction, hypothyroidism, hyperthyroidism, cerebrovascular accident, dyslipidemia, cancer, osteoarthritis, and arthritis. Multimorbidity was defined as the presence of two or more of these conditions. Nutritional status was assessed using the body mass index (BMI), categorized as underweight (BMI < 23.0 kg/m^2^), normal weight (23.0–27.9 kg/m^2^), overweight (28.0–29.9 kg/m^2^), and obesity (≥30.0 kg/m^2^) following the Pan American Health Organization guidelines [28]. Functional capacity was evaluated using the Short Physical Performance Battery (SPPB) which consists of three tests: balance, gait speed, and the ability to rise from a chair and sit down five times [29,30] and categorized into four levels: severe limitation (0–3 points), moderate limitation (4–7 points), mild limitation (7–9 points), and minimal limitation (10–12 points). Additionally, we included the following lifestyle variables: physical activity, defined as engaging in at least 150 min per week (equivalent to approximately 30 min per day), categorized as yes/no; smoking (current smoker, never smoked, or former smoker); and self-perceived health status (good, fair, or poor).

### 2.4. Data Analysis

The distribution of continuous variables was assessed using the Kolmogorov–Smirnov normality test, which indicated a non-normal distribution. Consequently, continuous variables were reported as medians and interquartile ranges (IQR), while categorical variables were presented as absolute and relative frequencies. Comparisons of food intake across NOVA groups were performed using the Mann–Whitney U test (sex) and the Kruskal–Wallis test (age group).

We used fractional probit regression models to assess the association between sociodemographic, economic, and health characteristics and the proportion of caloric intake from UPF (0–1 scale). Results are presented as mean marginal effects, multiplied by 100 to express the difference in percentage points of UPF intake between groups. Statistical significance was set at 5%. Data registration and storage were managed using REDCap 14.1.2 electronic data capture tool hosted at the Universidad del Desarrollo, Chile. All statistical analyses were performed with Stata software, version 16.1 (StataCorp, College Station, TX, USA).

## 3. Results

A total of 434 subjects were evaluated, predominantly female older adults (86.2%), with 58.5% aged 60–74 years and 76.3% residing in urban areas. Most participants lived in multigenerational households (57.8%) or alone (24.0%). Regarding education attainment, 56.0% had less than secondary education, whereas only 6.9% had higher education. Household income was below two minimum wages (MW) for 72.6% of participants, with 35.3% earning less than one MW. Most participants (94.5%) were affiliated with the public health system, whereas only 24 participants (5.5%) reported private health insurance (Table 1). 

Multimorbidity was reported by 79.3% of participants, and excess weight was highly prevalent (41.2% overweight, 35.0% obesity). Regarding self-perceived health status, 48.4% reported it as fair and approximately one-third as good. In terms of functional capacity, 54.4% had no limitations. Physical inactivity was common, with 55.1% engaging in less than 150 min of physical activity per week. Nearly half of the participants were current or former smokers (47.0%) (Table 1).

Table 2 summarizes the usual dietary intake by NOVA food groups according to sex and age. In the overall sample, the median daily energy intake was 554.9 kcal from unprocessed or minimally processed foods (group 1), 106.0 kcal from processed culinary ingredients (group 2), 289.4 kcal from processed foods (group 3), and 219.9 kcal from ultra-processed foods (group 4). These intakes represented 45.7%, 9.4%, 25.5%, and 16.6% of total daily energy intake, respectively. Women had lower median intakes than men across all NOVA groups, with statistically significant differences for group 1 (535.2 vs. 690.8 kcal, *p* < 0.001), group 2 (102.1 vs. 160.6 kcal, *p* < 0.001), and group 3 (278.3 vs. 399.1 kcal, *p* < 0.001). In terms of energy contribution, women had a lower percentage from group 2 (9.1% vs. 11.1%, *p* < 0.05). Across age groups, median energy intakes were relatively stable, with slightly higher contributions from processed foods (group 3) and UPF (group 4) among those aged ≥80 years. The contribution of the main ultra-processed food subgroups (NOVA 4) to total energy intake is presented in Supplementary Table S1. Packaged breads were the largest contributor to total energy intake (3.8%), followed by beverages (2.1%), processed meats (1.9%), and sweet bakery products (1.8%).

In fractional probit regression analyses, the crude models showed that rural residence was associated with a lower UPF proportion of energy, whereas completed secondary or higher education and private health insurance were associated with a higher UPF contribution; all three associations remained significant in multivariable models with similar direction and magnitude. Specifically, in adjusted models, rural residence was −3.76 percentage points (pp; 95% CI −6.34, −1.19; *p* = 0.004), secondary/higher education was +3.52 pp (95% CI 1.07, 5.96; *p* = 0.005), and private insurance was +9.06 pp (95% CI 4.12, 14.00; *p* < 0.001). No associations reached statistical significance for living arrangement, sex, age group, household income, food insecurity, BMI, NCD, smoking status, or physical activity in either the crude or multivariable models. Full crude and adjusted estimates are shown in Table 3.

## 4. Discussion

In this community-based sample of older adults from Chile’s Metropolitan Region, dietary intake was characterized by a predominance of unprocessed or minimally processed foods, followed by processed foods, while UPF contributed a smaller share of total energy intake. UPF intake was not associated with health or lifestyle indicators. Instead, a socioeconomic and place-based gradient emerged: rural residence was associated with lower UPF intake, whereas secondary or higher education and private health insurance were associated with higher UPF intake. These findings suggest that, in this population, UPF intake may be more strongly shaped by social and environmental factors than by current health status. Unprocessed or minimally processed foods contributed 45.7% of total energy intake, while UPF accounted for 16.6%. These findings are consistent with previous evidence from Chile and other countries. Cediel et al. reported that UPF contributed 18.3% (95% CI: 16.8–19.8%) of total energy intake among Chilean older adults (>65 years) [11]. Similarly, Silva et al. reported a comparable distribution in Brazilian older adults, with 61.2% of energy derived from unprocessed or minimally processed foods and 16.3% from UPF [31]. In many countries, national dietary surveys have identified an inverse association between age and UPF intake, with older adults generally consuming lower amounts than younger age groups [32], which may reflect generational differences in dietary habits and long-term exposure to industrialized food systems. Among UPF subgroups, packaged breads, beverages, processed meats, and sweet bakery products were the main contributors, suggesting that UPF intake in this population was mainly driven by a few commonly consumed products.

In our study, UPF intake was higher in urban areas compared to rural areas (−3.76%; 95% CI: −6.34 to −1.19). Similar patterns have been reported in Chile and other countries [12,13]. Geographical disparities between urban and rural areas may influence dietary patterns through differences in food availability, market access, and food environment. Urban settings often offer greater food diversity but are also characterized by a higher density of UPF and more obesogenic environment [15,32,33]. Conversely, rural areas may have lower availability of UPF but face logistical and economic barriers that limit food diversity and access to certain food groups [15].

A positive association was observed between socioeconomic indicators—particularly education and private health insurance—and UPF intake. This pattern contrasts with findings from several high-income countries, where higher UPF intake is often associated with lower socioeconomic status, but aligns with evidence from some middle-income settings, including parts of Latin America [6,32,33]. Individuals with higher socioeconomic status may also experience greater time constraints and a higher reliance on convenience foods, particularly in urban settings [34]. Nevertheless, this finding should be interpreted with caution given the small number of participants affiliated with the private health system in this sample (*n* = 24).

These findings should be interpreted within the context of contemporary food environments. Current dietary guidelines recommend prioritizing unprocessed or minimally processed foods and limiting UPF intake [35]. However, translating these recommendations into practice may be challenging in modern urban settings, where UPFs are widely available, convenient, and aggressively marketed [14,36]. These products are designed to be ready-to-consume, shelf-stable, and highly palatable, contributing to their rapid expansion in modern diets [14,36]. In addition, food cost and economic constraints may influence dietary choices, particularly among populations with limited resources [37]. In this context, UPF intake may reflect structural characteristics of contemporary food systems rather than solely individual dietary choices [34,36].

Beyond socioeconomic position, social and household contexts may also influence dietary practices in older age. Living arrangements and social support can shape food-related behaviors among older adults, as those living alone or with limited support may face barriers to maintaining regular meal preparation and balanced diet [38,39]. Despite this, no significant association was observed between living arrangement and UPF intake in this study.

We did not observe an association between UPF intake and health outcomes in this population. Several factors may contribute to this finding. The study focused on broader indicators of health status, including multimorbidity and self-perceived health, to provide a more comprehensive assessment of overall well-being among older adults. Older adults with lower UPF intake may have experienced reduced exposure to these products throughout their lives. In addition, the high prevalence of multimorbidity suggests that many individuals may have received medical advice encouraging healthier dietary practices. Given the cross-sectional design, causality cannot be inferred, and reverse causation cannot be ruled out. These factors may have attenuated potential associations between UPF intake and health indicators.

Evidence on the association between UPF intake and health outcomes among older adults remains limited. A recent systematic review identified only a small number of studies in individuals aged ≥60 years and reported associations between higher UPF intake and outcomes such as frailty, dyslipidemia, decline in renal function, and abdominal obesity [40]. Further research is needed to better understand how UPF intake relates to health trajectories in older populations.

This study has several strengths, including the use of 24-h dietary recalls with the multiple-pass method conducted by trained nutritionists. Usual intake was estimated using the Multiple Source Method, which accounts for intra-individual variability and provides more robust estimates. The NOVA classification system offered a standardized framework for characterizing dietary intake. Nevertheless, some limitations should be acknowledged. Given the cross-sectional design, causality cannot be inferred, and reverse causation cannot be ruled out. Although usual intake was estimated using the Multiple Source Method, the second 24-h recall was collected only in a subsample of participants, which may have affected the precision of the usual intake estimates. The sample size may limit the precision of association estimates, particularly in models including multiple categorical variables. To address this, some categories were collapsed in multivariable analyses to reduce the number of parameters and optimize degrees of freedom, although statistical power may remain limited for detecting modest associations. The sample was drawn from selected low-and middle-income municipalities in the Metropolitan Region and may not be fully representative of the national older adult population, particularly those living in remote areas or institutionalized settings. Therefore, the findings should be interpreted primarily as applying to community-dwelling older adults residing in the municipalities included in this study. Recruitment through community networks and senior clubs may also have introduced selection bias toward more socially active or healthier individuals. Additionally, the predominance of women in the sample (86.2%) may limit the generalizability of the findings to older men.

Although widely used, the NOVA classification system has been criticized because foods with heterogeneous nutritional profiles are categorized into only four processing groups [41]. To improve classification accuracy, detailed brand-level information was collected, and all items were classified independently by two researchers. Additionally, dietary intake was expressed as a percentage of total energy intake; therefore, foods or beverages with negligible or zero caloric contribution may be underrepresented.

Despite these limitations, this study provides relevant insights into dietary patterns among older adults in Chile. In the context of rapid population aging and evolving food environments, understanding the determinants of UPF intake is essential for informing public health strategies. Future research should focus on identifying the most harmful UPF subcategories, while policy efforts should strengthen access to healthy foods and promote food environments that make dietary recommendations practical and achievable in the Chilean context [42].

## 5. Conclusions

In this community-based sample of older adults from the Metropolitan Region of Chile, ultra-processed foods accounted for approximately 16.6% of total energy intake, while minimally processed foods remained the main contributors to dietary energy. UPF intake was associated with sociodemographic characteristics—particularly area of residence, education attainment and health insurance type—but not with health status or lifestyle factors.

Strategies such as strengthening local food systems, expanding access to fresh produce, and supporting community-based initiatives that encourage traditional dietary practices may contribute to healthier dietary patterns in aging populations. Addressing these multifaceted challenges is crucial to fostering an environment that supports healthier dietary choices and enhances the overall well-being of older adults in Chile.

## Figures and Tables

**Table 1:** Demographic and socioeconomic characteristics from the sample (*n* = 434).

Variables	*n*	%
***Sociodemographic and economic variables***		
*Sex*		
Female	374	86.2
Male	60	13.8
*Age group*		
60–69 years old	138	31.8
70–79 years old	208	47.9
≥80 years old	88	20.3
*Residential area*		
Rural	103	23.7
Urban	331	76.3
*Living arrangement*		
Lives alone	104	24.0
Lives with family (partner, children and grandchildren)	251	57.8
Other combinations	79	18.2
*Educational level*		
Less than secondary education	243	56.0
Complete secondary education	161	37.1
Higher education	30	6.9
*Total household income*		
Less than 1 minimum wage *	154	35.3
1 to 2 minimum wages	162	37.3
3 or more minimum wages	119	27.4
*Public health insurance system*	410	94.5
*Food insecurity probability* ^1^		
Moderate to severe, mean (SD)	11.5	0.28
Severe, mean (SD)	1.34	0.08
***Health-related variables***		
*Number of NCD*, median IQR	3	2–4
*Multimorbidity* (≥2 NCD)	344	79.3
*Nutritional status (BMI)* **		
Underweight	19	4.4
Normal weight	109	25.2
Overweight	73	16.9
Obesity	232	53.6
*Self-perceived health status*		
Good	136	31.3
Fair	210	48.4
Poor	88	20.2
*With any limitation in Physical Performance*	198	45.6
*Current or former smoker*	204	47.0
*Physically inactive*	239	55.1

* Reference minimum wage: $324,000 Chilean pesos (equivalent to approximately $365 USD in 2022). IQR: interquartile range; NCD: non-communicable chronic diseases. ^1^ Food insecurity probability was estimated using the FIES Rasch model (0–100%); higher values indicate a greater probability of food insecurity. ** BMI (body mass index) category cut-off points [28]: underweight < 23.0 kg/m^2^, normal weight 23.0–27.99 kg/m^2^, overweight 28.0–29.99 kg/m^2^ and obesity ≥ 30.0 kg/m^2^; SD: standard deviation.

**Table 2:** Usual dietary intake by NOVA categories: energy intake (kcal), and percentage of total daily energy intake (*n* = 434) general and according to sex and age group.

Variables	Nova System Classification							
	Group 1		Group 2		Group 3		Group 4	
	Med	IQR	Med	IQR	Med	IQR	Med	IQR
	**Energy intake (kcal)**							
*General*	554.9	281.5	106.0	99.7	289.4	189.3	219.9	229.5
*Sex*								
Female	535.2 *	264.4	102.1 *	93.8	278.3 *	184.0	214.9	219.2
Male	690.8	352.9	160.6	139.3	399.1	297.5	268.3	238.8
*Age group*								
60–69 years old	554.9	270.2	113.4	98.2	290.6	202.2	248.2	267.9
70–79 years old	542.9	283.7	103.5	105.5	281.9	185.0	208.8	207.5
≥80 years old	564.8	271.0	99.3	95.1	305.1	222.2	224.4	216.0
	**% of Total kcal/day**							
*General*	45.7	15.5	9.4	7.0	25.5	15.7	16.6	17.3
*Sex*								
Female	46.1	15.3	9.1 **	6.8	25.3	15	16.7	17.8
Male	44.4	16.8	11.1	8.6	26.9	17.7	16.6	15.2
*Age group*								
60–69 years old	45.2	15.1	9.9	6.7	23.9	16.9	16.8	20.5
70–79 years old	46.4	15.6	8.9	7.1	25.5	13.0	15.6	17.0
≥80 years old	45.8	15.8	9.1	7.1	27.7	20.0	18.5	15.9

Significance level * *p* < 0.001; ** *p* < 0.05. Med: median; IQR: interquartile range. Note: The usual intake was estimated using the MSM for each NOVA group. Consequently, the sum of the contributions from the four groups is close to but does not sum exactly to 100%.

**Table 3:** Crude and adjusted associations between sociodemographic, economic, and health variables and the proportion of energy intake from ultra-processed foods (*n* = 434).

Independent Variables	Crude			Multivariate		
	Mean Diff (%)	95% CI	*p*-Value	Mean Diff (%)	95% CI	*p*-Value
*Residential area*						
Urban	Reference			Reference		
Rural	−3.98	−6.45; −1.51	0.002	−3.76	−6.34; −1.19	0.004
*Living arrangement*						
Multigenerational	Reference			Reference		
Alone	0.55	−2.21; 3.31	0.697	0.87	−1.96; 3.69	0.547
*Sex*						
Male	Reference			Reference		
Female	−1.77	−4.70; 1.16	0.237	−0.40	−3.58; 2.78	0.805
*Age group*						
60–69 years old	Reference			Reference		
70–79 years old	−1.57	−4.30; 1.15	0.259	−1.94	−4.80; 0.92	0.183
≥80 years old	−0.52	−3.74; 2.70	0.751	−1.08	−4.58; 2.42	0.544
*Educational level*						
Less than secondary education	Reference			Reference		
Complete secondary or higher education	3.69	1.32; 6.07	0.002	3.52	1.07; 5.96	0.005
*Health insurance system*						
Public	Reference			Reference		
Private	9.57	4.77; 14.3	0.001	9.06	4.12; 14.00	<0.001
*Total household income*						
Less than 1 minimum wage	Reference			Reference		
1 to 2 minimum wages	−0.48	−3.26; 2.30	0.733	−0.28	−3.11; 2.56	0.849
3 or more minimum wages	−1.26	−4.18; 1.65	0.395	−1.96	−5.04; 1.12	0.212
*Food insecurity probability* ^1^	−1.63	−7.01; 3.76	0.554	−2.32	−7.53; 2.89	0.383
*BMI* (kg/m^2^)	−0.02	−0.23; 0.20	0.879	−0.01	−0.23; 0.21	0.933
*Number of NCD* ^2^	0.60	−0.12; 1.32	0.103	0.72	−0.06; 1.50	0.069
*Smoking status*						
Never smoker	Reference			Reference		
Current or former smoker	1.64	−0.69; 3.97	0.168	0.89	−1.44; 3.21	0.454
*Physical activity status* ^3^						
Active	Reference			Reference		
Inactive	−0.91	−3.24; 1.41	0.442	−0.34	−2.66; 1.98	0.775

CI: Confidence interval; BMI: body mass index; ^1^ Food insecurity probability was estimated using the FIES Rasch model (0–100%); higher values indicate a greater probability of food insecurity; NCD: ^2^ discrete numerical variable; ^3^ reference category: at least 150 min/week. Note: Effects are reported as mean differences in percentage points of UPF energy contribution (average marginal effects) from fractional probit regressions.

## Data Availability

The datasets analyzed during the current study are not publicly available but are available from the corresponding author on reasonable request.

## Supplementary Materials

The following supporting information can be downloaded at: https://www.mdpi.com/article/10.3390/nu18121899/s1, Supplementary Table S1: Contribution of ultra-processed food subgroups (NOVA 4) to total energy intake among older adults in Chile.
