# Supplementary material for: Sociodemographic, Economic, and Health Factors Associated with Ultra-Processed Food Intake Among Older Adults in Chile
Source: Nutrients. 2026 Jun 12;18(12):1899. doi: 10.3390/nu18121899 (PMC13304713; doi:10.3390/nu18121899)
Supplement: Supplementary file 1 [file nutrients-18-01899-s001.zip › nutrients-4364507-supplementary.pdf]

Supplementary Table S1. Contribution of ultra-processed food subgroups (NOVA 4) to total energy intake among older adults in Chile.

Ultra-Processed Food Subgroup	% Total Energy Intake
Packaged breads	3.8
Beverages[1]	2.1
Processed meats	1.9
Sweet bakery products	1.8
Flavored dairy beverages	1.2
Yogurt	1.1
Sweet cookies	1.1
Instant soups and broths	1.1
Sweet spreads and sauces	1.0
Savory crackers	0.9
Ready-to-eat meals	0.9
Chocolate, ice cream, and confectionery	0.9
Margarine	0.5
Dressings and savory sauces	0.5
Other UPF[2]	0.9

UPF: ultra-processed foods. [1]Beverages include sugar-sweetened soft drinks, fruit nectars, and industrialized fruit juices. [2]Other UPF includes breakfast cereals, snacks, distilled alcoholic beverages, nutritional supplements, and sweeteners, each contributing less than 0.2% of total energy intake.
